# The Role of the COVID-19 Crisis in Shaping Urban Planning for Improved Public Health: A Triangulated Study

**DOI:** 10.3390/ijerph20053804

**Published:** 2023-02-21

**Authors:** Koudoua Ferhati, Saliha Chouguiat Belmallem, Adriana Burlea-Schiopoiu

**Affiliations:** 1 AVMF Research Lab, Department of Project Management, Faculty of Architecture and Urbanism, Constantine 3 University, El Khroub 25000, Algeria; 2 Department of Project Management, Faculty of Architecture and Urbanism, Constantine 3 University, El Khroub 25000, Algeria; 3 Department of Management, Marketing, Business Administration, Faculty of Economics and Business Administration, University of Craiova, 200585 Craiova, Romania

**Keywords:** urban practices, public health, COVID-19, triangulated study, policy

## Abstract

This paper aims to assess the impact of the COVID-19 pandemic on the link between urban planning practices and public health. A triangulated study was conducted to gain a comprehensive understanding of the topic. The first phase consisted of semi-structured interviews with health and urban planning experts, which were analyzed with the aid of Artificial Intelligence tools. The second phase involved an on-site investigation in the city of Algiers, including a survey, site visits, and a thorough analysis of the master plan for land use and urban planning. The findings emphasize the critical importance of a comprehensive health-centric approach to city design, improved governance and management practices, community involvement, and political commitment to prioritize health in urban planning. Furthermore, the results proved a strong correlation between prioritizing public health in urban planning practices and residents’ satisfaction with the city’s response to the COVID-19 pandemic. In conclusion, it is necessary to consider public health as a priority in urban planning practices and as a need for all stakeholders to work towards a healthier and more equitable urban environment.

## 1. Introduction

The increasing rate of urbanization has raised concerns about urban areas’ environmental and health conditions. The challenges emphasize the need for safe distancing and precautionary measures in cities during and after the COVID-19 pandemic. A review of the existing literature and empirical evidence helps in understanding the impact of the COVID-19 pandemic on the relationship between public health and urban planning practices and guides the consideration of human health in city planning after the pandemic [1].

More information is needed to connect urban planning and public health in light of the consequences of COVID-19 in order to ensure adequate environmental conditions and create healthy cities, which has been an ongoing effort for nearly 30 years based on past research and practices, including sanitary surveys, park planning, and urban environment investigations [2,3].

A straightforward guide that outlines the factors impacting public health is necessary for policymakers and urban planners to understand the connection between shared urban planning and public health aspects [4]. The consideration of environmental and health factors as determinants of public health by all stakeholders has become crucial [5], especially in light of the COVID-19 pandemic. However, there need to be more explicit references in the literature and in the policy that addresses this topic. The impact of the pandemic on city health and well-being has been studied, and it has been found that changes in health and well-being were negative, especially in crowded neighborhoods [1]. The results show that urban planning practices need to be revised in the current circumstances, leading to changes in city management rules and urban planning, particularly in Algeria.

The pandemic has accelerated the shift towards remote work and online learning, significantly increasing sedentary behavior and screen time, which can negatively affect mental and physical health. Additionally, with the outbreak of COVID-19, cities worldwide have faced significant disruptions to daily life as governments implemented measures to slow the spread of the virus. These measures included the closure of schools, offices, and businesses, the cancellation of cultural events such as concerts and tradeshows, and bans on gatherings [6,7], which showed the importance of access to green and outdoor spaces for physical activity and mental well-being after passing this challenging era.

Three years after the COVID-19 pandemic, it is important to study the long-term impact of urban planning practices on public health. The pandemic has changed our understanding of what makes a city healthy; the focus is on access to healthcare facilities, clean air, and water, and adequate public spaces. Understanding how the pandemic has altered our perception of a healthy city is essential. Conducting a study on the impact of post-COVID-19 urban planning practices on public health can provide valuable insights into how urban design can promote healthy behaviors and reduce health disparities.

This paper raises the challenge of comprehending the effect of the pandemic on urban planning behavior and the relationship between urban planning practices and public health to offer clear guidance to engineers and policymakers. This study is innovative in its examination of the context of northeastern Algeria and its focus on identifying opportunities for improvement in urban spaces through the evaluation of green spaces, sewage treatment technologies, and the role of urban planners in promoting healthy cities. It bridges the gap between macro health-oriented strategies and actual managerial practices by examining the practical implementation of policies to enhance public health.

## 2. Theoretical Framework and Hypotheses Development

To understand how urban public health and urban planning might be affected by COVID-19 consequences, we need to have a clear understanding of the main elements in direct contact with the individual’s health, namely their daily activities, macro agendas affecting their lifestyle, and also the rudiments that shape the environment surrounding these individuals. Every urban element, idea, or practice has associated health issues [6].

According to Lazuardi et al. [8], there is a close relationship between health and urban planning; they highlight the role of public health indicators in cities in improving citizens’ psychological conditions. Giacoman and colleagues [9] found that urban planning practices such as increasing access to green spaces and promoting active transportation can positively affect public health outcomes, such as reducing rates of obesity and improving mental health. Braubach et al. [10] and EL helou [11] suggest that certain urban design elements, such as mixed land use and pedestrian-friendly streets, can improve public health outcomes, such as reducing rates of obesity and promoting physical activity.

Cain and colleagues [12] support their study that urban design practices that encourage walking and biking, such as providing safe and accessible pedestrian and bike infrastructure, can increase physical activity levels and reduce rates of obesity and other chronic diseases. In addition, Rowe and colleagues [13] found that some aspects of the built environment, such as access to green spaces and social cohesion, can positively affect mental health outcomes, such as reducing rates of depression and anxiety.

Many researchers [14,15,16] agree that there are different levels of influence, explained as follows:

First, at the sanitary level, the rapid and dispersed urban growth in 19th-century cities due to fast industrialization practices and lack of epidemiological considerations in urban planning led to unhealthy living conditions and the importance of considering the impact of architectural spaces on public health [17]; the environment of the urban periphery can greatly impact the state of public health [18], which does not consider the illnesses’ special requirements.

The characteristics of the urban space are linked to negative health outcomes, such as health issues, disability, mental illness, and even mortality [19]. In addition, evidence shows that air and noise pollution from traffic, poor sanitary systems, and residential exposure to high traffic are shown to have negative health effects, including asthma, lung development, allergies, sleep disturbance, children’s cognitive development, and increased risk of hypertension and coronary heart disease [20,21,22,23].

Based on the above considerations, it is hypothesized that:

**Hypothesis** **(H1):** *Implementing urban planning practices that promote improved sanitary systems after COVID-19 significantly positively impacts public health outcomes.*

The second level of influence on public health in urban environments is related to governance and the decision-making processes in urban planning. The design and planning of neighborhoods and cities can significantly impact residents’ mental and physical well-being. Jutraz and Kukec [24] suggest that neighborhoods can impact individual health by shaping behaviors and limiting resources, with proper facilities and resources such as parks and recreational centers promoting healthy behaviors and improving public health outcomes. 

The availability of green spaces can also impact public health. For example, industrialization and technological advancements in the 19th and 20th centuries negatively impacted green space availability, which led to a decline in urban green spaces and negatively affected water quality and quantity [16].

Furthermore, modern planning practices have often ignored the importance of greenery in cities [25], but recent studies suggest that access to green spaces can positively impact mental and physical health [26,27]. 

Based on these considerations, it is hypothesized that:

**Hypothesis** **(H2):** *Implementing urban planning practices that prioritize residents’ mental and physical well-being post-COVID-19 significantly impacts public health outcomes*.

Policy implications in urban planning could be observed and evaluated in programs and initiatives provided through the last decade [28,29]. The Algerian government has shown a commitment to sustainable development over the years. Moreover, the government commitment was solidified in the aftermath of the United Nations Conference on Environment and Development in 1992, which emphasized the importance of sustainable policy orientations that prioritize the well-being of citizens and their harmonious relationship with nature [30,31]. The high council for the Environment and Sustainable Development was established in 1994 to oversee environmental protection and sustainable development efforts [32]. However, this council faced criticism for its lack of efficiency [33]. In 2002, the National Plan of Action for the Environment and Sustainable Development was created to improve the quality of the environment and urban life quality. The decree n°03-10 of 2003 established the fundamental principles and regulations of environmental management and encouraged public participation in environmental protection efforts [34]. In 2006, the Town Orientation Law was approved by the parliament to establish specific provisions for a sustainable development policy, and an international competition was launched to create a sustainable urban master plan for Algiers. The winning project, “Making Algiers a Green Metropolis of the Mediterranean”, will be implemented in 2035. 

In the regulation context, in response to the critical environmental issues resulting from the intensive development strategy since the post-independence period, the Algerian government has developed a National Environmental Strategy. This strategy involves the creation of effective policies for the protection, enhancement, and regulation of environmental-related usage at various scales and in different management contexts, including land management, waste management, natural resource management, and energy control through the implementation of various laws and regulations, such as the 1983 Law on Environmental Protection, the 1987 Law on Territorial Planning, the 1990 Law on Land orientation, the 2001 Law on Sustainable Territorial Development, the 2002 Law on Coastal Protection and Valorization, the 2003 Law on Environmental Protection in the Context of Sustainable Development, and the 2004 Law on Major Risk Prevention, as well as laws on energy control and the promotion of renewable energy [35]. Finally, we hypothesized that:

**Hypothesis** **(H3):** *Policy initiatives and programs that support sustainable development and improve the urban environment significantly impact public health outcomes.*

**Figure 1:**
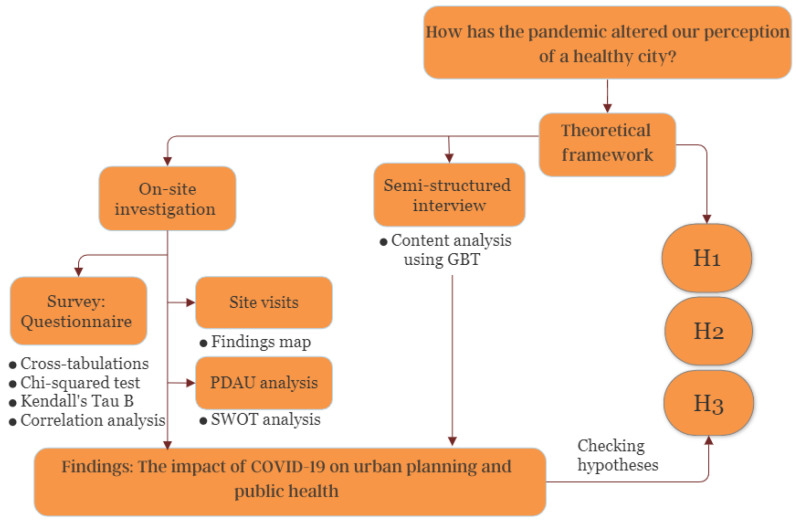
Model of the study. Source: authors.

Based on the previous theoretical framework study [36,37], discussing the different elements and issues related to urban planning that might affect public health and the development of the three research hypotheses, we propose in Figure 1 the following study model:

## 3. Materials and Methods

A triangulated study that employs a mixed methodology approach was conducted in two phases. In the first phase, qualitative methods, including semi-structured interviews, were used to gain an in-depth understanding of the relationship between urban planning and public health after COVID-19, as viewed by health and urban planning professionals. The second phase involved a combination of quantitative and qualitative methods, including a survey, site visits, and document analysis to supplement the findings of the first phase. The triangulated study is a research strategy that employs multiple data sources, methods, theories, and/or researchers to address a research question, enhance the validity and credibility of the findings, and reduce the presence of any research biases in this paper. 

### 3.1. Planning and Carrying out the Interview

There were three main phases for conducting the interview: the planning, the actual carrying out, and the analysis of the empirical material [38].

In the interview planning process, we had to make sure that the method was suitable to the study’s objectives and that it could answer the research question. According to the participatory design processes [39] and the evaluation and action research, the choice of research methods is always linked to the scope and objective of the study [40]. Since the primary study objective is to understand and have an impact on relations based on professional experience, local practices, or human constructions of the meaning of public and urban planning practices, the qualitative interview should be considered [41].

**Figure 2:**
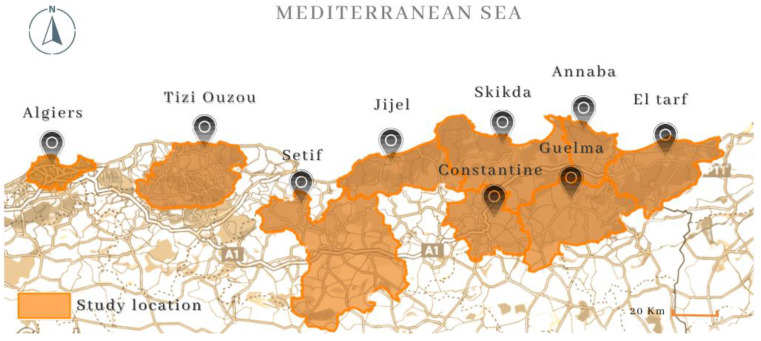
Study location map. Source: authors.

After ensuring that an in-depth interview is a suitable method for the research, it is time to ask the important questions: who, where, and what should be the topics to be included in the interview? It is essential to pick suitable informants when the research methodology is based on personal records [42]. The choice of participants for the semi-structured interviews was based on the criteria of expertise and relevance to the research topic. Managers, doctors, and engineers from urban planning and health administration were selected as participants because they have professional experience and knowledge in the areas of urban planning, public health, and governance and are therefore well-suited to provide valuable insight and perspectives on the northeastern part of Algeria: 09 wilayas, as shown in Figure 2.

The nine wilayas studied in the research showcase a diverse socio-economic profile, reflecting Algeria’s cultural and geographic richness. Algiers serves as the political and economic center, while Tizi Ouzou is a significant city in the Kabylie region known for its coastal forests. Constantine is the capital of eastern Algeria, and a commercial hub such as Annaba boasts large steel, food, and port industries. Despite having well-developed healthcare systems, these metropoles faced significant health challenges during the pandemic, including overcrowding and stress on healthcare systems. Guelma, Setif, Skikda, and Jijel are mixed-activity wilayas with a smaller population featuring agriculture and industry. Finally, El Taref is a smaller coastal city with a lower population size, agricultural economy, and cultural heritage. Before the pandemic, these cities faced complex public health issues such as air and water pollution and insufficient healthcare facilities and staff.

The sampling phase for the interviews was guided by the Hagaman and Wutich method [43], which recommended around 20 participants to identify new themes and saturate categories; ultimately, 12 interviews were conducted due to the non-availability of some participants. We designed the semi-structured interviews based on the themes from the theoretical framework: sanitary system, spaces management, and policy implementation-related questions (25 questions), enabling openly valid and reliable answers (Appendix A). The interviews were done in person and via an online platform (Google meet and zoom) between 7 September 2022 and 19 December 2022, depending on the ability of the participants and authors. The study conducted in-depth qualitative interviews with 12 participants, with varying lengths between 36 and 48 min; anonymity was ensured and consent was obtained before discussing public health, urban managerial planning strategies, and potential impacts.

The thematic analysis of the categorized questions was conducted using Generative Pre-trained Transformer (GBT) to identify patterns and trends in the interview data, which generated three main themes:

1. Sanitary system and epidemiology.
2. Healthy city, management, and green spaces implementation.
3. Health integration in urban planning policy.

The themes were analyzed to extract qualitative data.

### 3.2. On-Site Investigations

The methodology involved a mixed-method approach. We used a combination of a survey, site visits, and an Urban Development Master Plan (PDAU) analysis.

A questionnaire (Appendix B) was designed using a 5-point Likert scale based on the theoretical framework and the semi-structured interview perspectives to conduct the survey. In addition to the survey, we conducted a site visit to investigate the main declared initiatives and to observe ongoing projects and initiatives. Then we analyzed Algiers city PDAU horizon 2035 to understand the urban planning practices from all perspectives.

- The survey:

Sampling and case study:

The respondents were the citizens of Algiers, the capital and largest city of Algeria, which had undergone significant changes in its urban conditions before and during the COVID-19 pandemic. Pre-pandemic, Algiers was characterized by a rapidly growing population, an estimated 4.5 million residents in 2020, and a bustling commercial center. However, like many large cities, Algiers faced challenges related to the lack of appropriate accessible public spaces, sewage and water pollution problems, air pollution due to of the high industrial concentration and the considerable number of car users in the center of the city, and overcrowding in certain areas. During the pandemic, the city saw a significant decrease in population mobility and economic activity, with measures such as lockdowns and social distancing regulations implemented to slow the spread of the virus.

A random sampling method was used to select 200 participants from the population, and we identified 112 valid and complete questionnaire responses. The sample was chosen to be representative of the population in terms of demographic characteristics such as age, gender, occupation, and education level.

Data collection:

The survey was administered in person to the selected participants. We made sure to explain the purpose of the survey and ensure that the participants understood the questions before they began answering.

Data analysis:

A categorical data analysis was conducted to examine the relationship between variables. The analysis included the use of cross-tabulations, the chi-squared test of independence, and Kendall’s Tau B correlation analysis. 

The cross-tabulation, also known as contingency tables, was used to determine the variables’ frequency distribution and test the relationship between two categorical variables. The chi-squared test of independence was used to determine whether the frequency distribution of one categorical variable is independent of the frequency distribution of another categorical variable. Finally, Kendall’s Tau B correlation analysis was used to determine the association between two ordinal categorical variables. This method allowed us to examine the relationship between different variables in each hypothesis to provide insight into their association and dependence.

b. Site visits:

In order to validate the findings from the survey and to investigate the main declared initiatives and ongoing projects in Algiers that reflect the COVID-19 effect on urban behaviors in the city, we conducted a series of site visits. To summarize the findings of our on-site visits, we employed a statistical approach by calculating the mean completion percentages for each initiative. This was achieved by summing the completion percentages and dividing them by the number of initiatives, as detailed in Table 4. Our estimation of the progress of each initiative was based on a combination of our judgment and observed documents, where we observed the ongoing activities and their progress, and then conducted informal interviews with government officials and members of civil society to gather additional information about the status of initiatives and projects’ execution; we also analyzed available data and documents when needed. These sources of information were used to estimate the completion percentages for each initiative and record them in our findings.

c. Document analysis:

In this step, we analyzed the main urban planning tool for Algiers: PDAU horizon 2035 [44]. We examined and categorized the content from the presentation and components, pressures, opportunities, risks, and emerging problems, and synthesized the key points in a SWOT matrix used for identifying and analyzing an initiative’s strengths, weaknesses, opportunities, and threats.

## 4. Results

### 4.1. The Semi-Structured Interview

**Figure 3:**
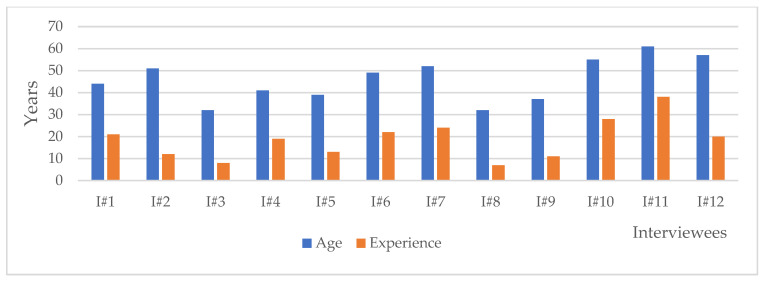
Age and experience. Source: authors.

For the purpose of analysis, a code was assigned to each of the twelve interviewees as follows: I#1, I#2, I#3, I#4, I#5, I#6, I#7, I#8, I#9, I#10, I#11, and I#12. In Figure 3, the variables of age and experience are presented on the Y axis (in years), with interviewees I#1 to I#12 shown on the X axis. The age range varied between 32 to 61 years, with a mean age of 45.83, and most of the interviewees were in the age range between 37 and 52. The experience variable ranged from 7 to 38, with a mean of 18.58. Similarly, when we look at the frequency of the experience variable, most interviewees had between 11 and 24 years of experience, with only one interviewee at each level of experience. Males (9 males and 3 females) were predominant in the gender distribution among the interviewees because of the specificity of the field of activity. This data provides a general overview of the demographic characteristics of the interviewees and can be used to understand the background and qualifications of the interviewees. 

In this phase, 12 interview answers were analyzed to investigate the perceptions of urban planners, managers, and public health professionals on the impact of managerial urban planning practices on public health. The results were grouped into three main themes.

#### 4.1.1. Sanitary System: Sewage, Toxification, Trash, and Epidemiology Theme

The results from the analysis of the interviews from the first theme are presented and interpreted in Table 1.

#### 4.1.2. Healthy City, Management, and Governance Theme

The results from the analysis of the interviews from the theme management, governance, and leadership skills for the identification of the healthy city concept are presented and interpreted in Table 2.

#### 4.1.3. Health Integration in Urban Planning Policy Theme

The results from the analysis of the interviews from the third theme, integrating health concepts in the urban planning process, are presented and interpreted in Table 3. 

### 4.2. On-Site Investigation

The demographic analysis of the sample of 112 survey respondents provides insight into the characteristics of the population surveyed. The gender distribution of the respondents is notably skewed, with 67% identifying as male and 33% identifying as female. The respondents’ ages range from 36–55 years old, and the preponderant are those between 36–45 years old (28.6%).

#### 4.2.1. The Survey

Regarding occupation, the sample comprises 47.3% of respondents working in the public sector, 15.2% are from the private sector, 16.1% are retired, 6.3% are self-employed, and 15.2% are students.

Regarding educational attainment, the sample is relatively well-educated, with 55.4% of respondents holding a university degree, 13.4% having post-graduate education, 20.5% having a high school education, and 10.7% having less than a high school education.

Categorical data analysis:

Before performing categorical data analysis in SPSS, we ensured that the data met specific requirements. Firstly, the data should be in a format appropriate for the tests. This typically means that the data should be in a categorical format; in our case, it is ordinal. Additionally, it is important to check for missing data and ensure no missing values in the analyzed variables.

The test results on satisfaction with the city’s response to COVID-19 with the city’s public health system accessible to all residents. The case processing summary shows no missing data, with all 112 respondents included in the analysis.

The distribution of responses for both variables, with the chi-square tests indicating a statistically significant association between the two variables (*p* < 0.001) through Pearson Chi-Square, Likelihood Ratio, and Linear-by-Linear Association.

The symmetric measures of Kendall’s tau-b and Spearman Correlation were used to examine the ordinal association between the two variables and both measures yielded high values of 0.698 and 0.772, respectively, which are statistically significant (*p* < 0.001) and indicate a strong ordinal association between the two variables. The data suggest a statistically significant and robust association between city residents’ satisfaction with the city’s response to COVID-19 and their perceptions of the city’s public health system’s accessibility and preparation for future epidemics.

The test results addressed satisfaction with the city’s response to COVID-19 and with the city’s public health system’s preparation for future epidemics. The crosstab table shows the count of responses for satisfaction with the city’s response to COVID-19 and the city’s public health system preparation for future epidemics. The chi-square tests indicate a significant association between these two variables (with a *p*-value less than 0.001). The symmetric measures (Kendall’s tau-b, Spearman Correlation, and Pearson’s R) also show a strong positive correlation between the two variables, with values of 0.741; 0.816; and 0.789, respectively. These results suggest that as the city’s public health system prepares for future epidemics, satisfaction with the city’s response to COVID-19 also increases.

For the second research hypothesis, the results of the tests addressed satisfaction with the city’s efforts to promote a healthy lifestyle with the city’s management level for green spaces. The first set of results is a crosstabulation and chi-square analysis examining the relationship between satisfaction with the city’s response to the COVID-19 pandemic and the city’s public health system’s preparation for future epidemics. The chi-square tests indicate a statistically significant association between the two variables (*p* < 0.001), with the largest expected count being 8 and the smallest expected count being 1.13. Additionally, the symmetric measures (Kendall’s tau-b, Spearman correlation, and Pearson’s R) all indicate a strong correlation between the two variables, with Kendall’s tau-b, spearman correlation, and Pearson’s R coefficient of 0.741; 0.816; and 0.789, respectively.

The second set of results is a crosstabulation and chi-square analysis examining the relationship between satisfaction with the city’s efforts to promote a healthy lifestyle, the city’s management level for green spaces, and the importance of green space accessibility. The chi-square tests indicate that there is a statistically significant association between the two variables (*p* < 0.001), with the largest expected count being 47 and the smallest expected count being 0.64. Additionally, the symmetric measures (Kendall’s tau-b, Spearman correlation, and Pearson’s R) all indicate a strong correlation between the two variables, with Kendall’s tau-b, spearman correlation, and Pearson’s R coefficient of 0.836; 0.882; and 0.916, respectively.

The crosstab shows the counts of responses for the two variables “satisfaction with the city’s efforts to promote a healthy lifestyle” and “the importance of green spaces accessibility” on a 5-point Likert scale. The chi-square tests indicate a significant association between the two variables, as the *p*-values are less than 0.001 for all three tests (Pearson Chi-Square, Likelihood Ratio, Linear-by-Linear Association). The symmetric measures section provides information on the strength and direction of the association between the two variables, with all three measures (Kendall’s tau-b, Spearman Correlation, Pearson’s R) showing a strong positive association (values ranging from 0.699 to 0.804) and significant *p*-values (less than 0.001). The N of valid cases is 112. Overall, the results suggest that there is a significant positive association between satisfaction with the city’s efforts to promote a healthy lifestyle and the importance of green space accessibility.

Results for statistical analysis of the third hypothesis show the frequency of responses for the two variables being studied: “satisfaction with health and safety measures implemented in the city after COVID-19” and “the city’s urban planning policies level promoting health and well-being”. The chi-square tests determine if there is a significant association between the two variables. The Pearson Chi-Square, Likelihood Ratio, and Linear-by-Linear Association tests were significant, with *p*-values less than 0.001.

The symmetric measures are used to quantify the strength of the association between the two variables. The ordinal-by-ordinal measures, Kendall’s tau-b, and Spearman Correlation indicate a strong association between the two variables, with values of 0.811 and 0.862, respectively. The interval-by-interval measure, Pearson’s R, also indicates a strong association with a value of 0.844.

The results of the following two variables show a significant association between satisfaction with the health and safety measures implemented in the city after COVID-19 and the effectiveness of management addressing public health concerns related to COVID-19. Moreover, the *p*-values are <0.001 for all three chi-square tests (Pearson Chi-Square, Likelihood Ratio, Linear-by-Linear Association) and all three symmetric measures (Kendall’s tau-b, Spearman Correlation, and Pearson’s R). The chi-square tests and symmetric measures provide measures of the strength and direction of the association between the two variables. For example, Kendall’s tau-b, Spearman Correlation, and Pearson’s R coefficient are 0.889; 0.912; and 0.908, respectively, all of which are close to 1 and indicate a strong positive association between the two variables.

#### 4.2.2. On-Site Visit

**Figure 4:**
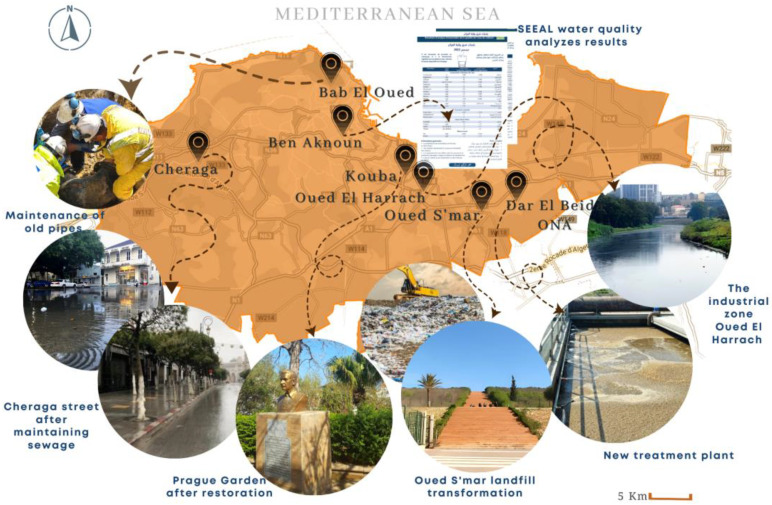
Key initiatives after COVID-19 in Algiers. Source: authors.

The data concerning the public health situation in the city of Algiers after COVID-19 were collected from the interviews and revealed ongoing efforts in various domains, including sanitary systems and epidemiology, water quality, toxic exposure risk, waste management, efficient land utilization, and green space administration. To validate these findings, a series of site visits were conducted, including the examination of VRD (Roads and various networks) plans, observations of construction sites, systematic analysis of potable water, assessments of pipeline conditions during precipitation, interactions with corporations and citizens, and inspections of public parks. The key initiatives spotted are presented in Figure 4.

The findings indicate that the efforts to prevent sewage mishaps and maintain the sewage network are underway and currently stand at 40% completion, which included operational measures to clean sewer systems, address malfunctioning systems, and repair existing breakdowns. The quality of water was found to comply with established standards. Upgrading outdated pipelines and the maintenance of access points is ongoing at 60% completion. The measures implemented include cleaning and maintaining 696,751 gutters and stormwater channels, essential for proper water drainage, and removing over 400,000 tons of mud and waste. The mitigation of toxic exposure risk is underway at 30%. The collection and disposal of waste are being handled by a private company and the government and are currently at 40% completion. The incorporation of new technologies in waste management is underway at 30%.

Additionally, the transformation of the Oued S’mar public landfill into a public garden has been fully completed. Furthermore, the renovation and creation of green spaces across all districts of Algiers are in progress and stand at 20% completion, although we found gaps in the availability and accessibility of green spaces in different parts of the city. Finally, the Bainam forest tree plantation initiative is underway at 90% completion after its severe degradation over the last ten years due to several reasons such as fires and natural causes.

When discussing with citizens during our investigation, we also observed the launch of awareness campaigns in partnership with members of civil society, which focus on the role of citizens in preserving the environment and enhancing public health conditions. As a result, on average, 58% of the areas inspected have been found to validate the initial findings. The detailed steps of the investigation are presented in Table 4.

#### 4.2.3. Document Analysis: PDAU Horizon 2035

**Figure 5:**
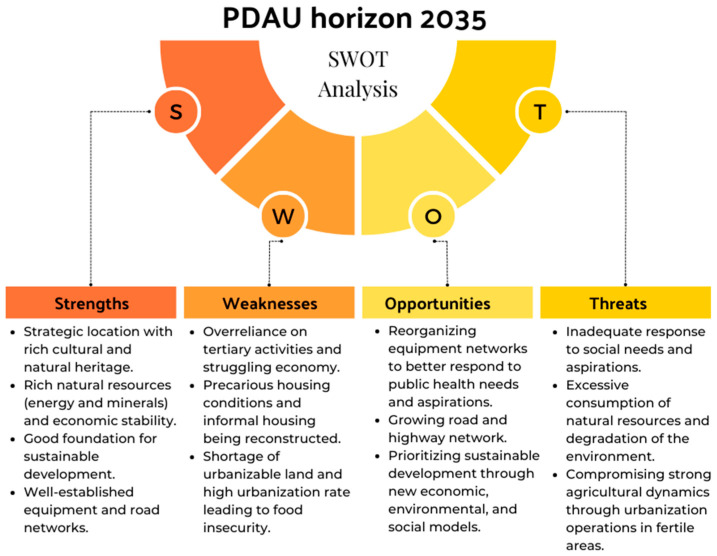
SWOT matrix for PDAU horizon 2035. Source: authors.

A rigorous diagnostic method, accompanied by continuous communication with local authorities and sectoral institutions, was ensured to deeply understand the territory and its specific realities for making this document analysis that englobes all the parts of the PDAU for the identification of the conditions, options, and principles established by the new territorial management tool that should guide and structure the development of the territory of the wilaya of Algiers for the next 12 years. We synthesized the main strengths, opportunities, weaknesses, and threats from the analysis in a SWOT matrix as shown in Figure 5 below.

Since independence, Algiers has faced a demographic explosion which resulted in infrastructure construction without regard for coherence, sustainability, or public health considerations. To address this issue, the Algerian government has decided to develop a strategic plan for the city (in 2016) to become a flagship city, safe for its residents, and competitive for economic agents, with good sustainable urban governance.

Therefore, the PDAU analysis is structured as follows:

Presentation and Components:

The master plan of the PDAU in Algiers has six pillars that form the foundation for the future of Algiers, intending to become a reference in the Mediterranean and the world:

- Economic Development, Competitiveness, and Employment.
- Opening the city to the World and Internationalization.
- Territorial Cohesion, Social Cohesion, and Housing.
- Environment, Protection, and Enhancement.
- Territorial Model.
- Governance.

These pillars are materialized through 82 key projects corresponding to concrete intervention proposals and provide substance to the territorial model proposed in the master plan. The key projects include the Port of Algiers, the Logistics Corridor of Ezzouar and Bab Ezzouar, the Hussein Dey/Mohammedia Seashore, the Central Station of Algiers, the Complementary Network of Industrial and Service Activities, the Faculty of Medicine, the Faculty of Law, the Douera Stadium, the participation of the private sector in residential production, the improvement of conditions for businesses and the banking sector, the revitalization and commercial upgrading of Algiers, the revitalization of urban core areas and agricultural activity, El Harrach Park and El Hamiz Park, the regional energy network of Algiers, the regional communication systems, e-wilaya strategy, the management of public space, and several ports and universities.

The Pressures:

The report analyzes the current pressures faced by Algeria. The economy relies heavily on tertiary activities and is struggling with a 20% unemployment rate and a growing informal economy. The country is also facing an economic crisis and a slow population growth rate, with 70% of its population under the age of 40, and an increasing demand for housing, equipment, and infrastructure. Housing conditions are precarious, with informal housing being reconstructed, and there is a new migration phenomenon due to factors such as climate change, economic challenges, and geopolitical issues.

Urbanization is a significant challenge, with a 94% urbanization rate and a shortage of urbanized land leading to food insecurity. The country also faces water stress, with only 160 L per capita per day, and energy security risks, with an average of 3150 kWh per household per year. Finally, climate change poses significant risks, including a temperature increase of +2° by 2030, a sea level rise of +16 cm, a decrease in rainfall of −10–15%, increased drought, and erosion.

Problems:

Algeria is facing urbanization issues, excessive consumption of natural resources, degradation of the environment, and transportation problems. The Algerian economy faces structural weaknesses such as a lack of diversification and integration into the global economy, dependence on international markets (hydrocarbons and agriculture), low human resources qualifications, technological innovation lag, and a lack of infrastructure to support economic activities. The challenges of sustainable development, based on the three pillars of efficient economic development, social equity, and environmental sustainability, are constantly being postponed due to various pressures.

Opportunities:

However, with its excellent geographical location, Alger enjoys a rich cultural and natural heritage. The country and its capital are increasingly attracting foreign direct investment, and with its rich natural resources (energy and minerals) and economic stability, Algeria has a favorable industrial potential supported by public investment in infrastructure to support economic activity. The youth of its population provide Algeria with a promising future and a good foundation for economic dynamism, provided it is trained, educated, and qualified. Therefore, it is important to prioritize sustainable development through the new economic, environmental, and social models stated in various national, regional, and sectoral plans to ensure a sustainable future. The various communities and sectors need to use all the tools developed at the highest level to address the existing problems in resource protection, environmental protection, and territorial planning. Agriculture is a valuable economic resource that should be preserved and developed sustainably, considering the climate and soil richness. Technological innovations should be introduced in agricultural production and processing to improve the profitability of the sector and the utilization of its resources. The export potential of agricultural products opens up prospects for this sector, especially for specific products with natural comparative advantages and growing demand. Urbanization operations should maintain strong agricultural dynamics, mainly when they spread to fertile areas of the territory. Although the equipment networks are generally well organized in terms of quantity and territory, they still need to be reorganized to better respond to social needs and the aspirations of the population and allow the emergence of a polycentric urban system. The road and highway network, well established in much of the wilaya of Alger territory, is overgrowing due to a vast construction and expansion program.

Mobility:

The public transportation network is a crucial aspect of the mobility sector, including different modes, their new paths, redirections, and requirements that have been highlighted in the PDAU as a crucial tool for territorial management and provides a vision for the city’s mobility plans involving stakeholders: first highways and expressways—equipped with characteristics to ensure optimal mobility and safety; main arteries—which form the leading urban network, complementing the first level. On these arteries, the traffic is mostly passing, and the first function is to serve the main generators and development poles of the region; secondary arteries have a similar function to the previous level but with less importance from a geographical point of view; and collector streets that locally important roads at the commune level. In complement to the road network, the mobility system envisages a three-level parking subsystem: park-and-ride lots, street parking (paid and non-paid), and off-street parking—accompanying parking lots. Finally, the mobility system considers the soft mode networks, i.e., the pedestrian and bike path networks.

Risks:

The plan considered managing earthquakes and reducing seismic risk in the region, focusing primarily on the seismic vulnerability of buildings. Measures to improve seismic resistance in new construction, renovation of existing buildings to increase their resistance, restrictions on construction in high-risk areas, and preparation for earthquakes by securing unstable equipment, are all part of the plan. Additional studies may be conducted for high-risk seismic areas to further understand the risks and determine appropriate technical solutions, as mentioned in the document. The plan also includes measures to improve infrastructure, such as increasing open spaces and improving road connectivity. In addition, the plan provides guidelines for reducing the risk of landslides and falling rocks. The laws related to urban planning and development in Algeria, such as Law No. 90.29 and Law No. 04-05, also address these issues and identify high-risk areas and implement measures to mitigate the risks.

Highlights and Critics:

From the observed urban planning practices expressed in the document, it is evident that hard work has been dedicated to the document’s execution, which included many positive points in fields of land use, sustainable resources alternatives implementation, and mega projects planning, but some points present a weakness in the plan’s strategies. First, we are continuing with the existing urban development plans (POS—land use plan), while we should be transitioning to more comprehensive urban development projects (Projets Urbains de Développement). Second, we are limiting our focus to the wilaya, while the metropolitan area is already a functional entity and the Mediterranean network is becoming more important. Third, we are neglecting external pressures such as the climate, geopolitics, and resources, which significantly impact development. Fourth, we are comparing ourselves to other countries, while we should first focus on internal benchmarks between the different communes in Algiers. Finally, while the PDAU covers various aspects of seismic risk mitigation, it does not include any considerations for pandemics or other similar health crises. In today’s world, where pandemics like COVID-19 have profoundly impacted communities, it is important to include public health considerations in any risk mitigation plan. Failure results from a limited response to such crises and leaves communities vulnerable.

## 5. Discussion

The first theme of healthy cities and sanitary systems encompasses the issues of sewage, toxic materials, waste management, and epidemiology, which are of crucial importance in urban planning practices. The opinions and insights shared by the interviewees shed light on the current state of the sewage systems, industrial zones, and the associated risks to public health. The consensus among the interviewees is that there is a need for collaboration between the conservation and public health sectors to address these issues and to maintain public health and cleanliness in cities. While some interviewees believe that the risk of exposure to toxic materials and chemical mixtures in urban environments is relatively low, others consider it a significant cause of concern. In addition, the rapid development of industrial zones in some areas of Algeria presents difficulties for control agencies monitoring toxic waste data, thus emphasizing the importance of a well-functioning waste management system to ensure public health.

In addition, the interviewees emphasize the significance of preventive strategies in urban planning, particularly in light of the ongoing pandemic. They advocate for a shift in traditional planning approaches towards a more holistic, health-centric approach to city design to ensure public health and safety in urban areas. The importance of staying informed about new waste management technologies and their impact on public health is also noted.

In conclusion, the insights shared by the interviewees provide evidence for the critical role of the sanitary system in promoting public health in urban areas. Furthermore, their views highlight the need for a concerted effort between various stakeholders and the government to address these pressing issues.

Examining the interviewees’ viewpoints about the impact of urban planning practices on public health reveals several salient themes. Firstly, there is a perception of a disconnect between overarching health-oriented strategies and their implementation at the city level, requiring improved governance and management practices to bridge this gap. Secondly, the interviewees recognize the importance of collaboration between various sectors to achieve the goals of healthy cities, highlighting the need for clear plans, budgets, and a continuous approach.

Additionally, the qualifications and training of healthy urban planners emerged as a point of contention among the interviewees, with some perceiving a need for sustainability and green city expertise. In contrast, others acknowledged the vital role of qualified managers and urbanists in achieving healthy city objectives. The interviewees also emphasized the crucial role of community involvement in realizing healthy city goals, highlighting successful examples in Algeria, where cooperation and collaboration between the government and citizens have been instrumental in advancing the healthy city concept.

In conclusion, the collective views of the interviewees demonstrate the significance of urban planning practices that prioritize residents’ mental and physical well-being in achieving improved public health outcomes. In addition, the need for competent governance, effective managerial decisions, and community involvement is emphasized, further underlining the importance of prioritizing health in urban planning.

The participants highlighted the importance of taking a comprehensive approach to improving the health and well-being of urban communities. Moreover, they acknowledge the current limitations in urban planning, and the need for increased efforts to address the identified gaps in urban health. Also, they emphasized the need for a shared vision among stakeholders, policy decision-makers, and partners to achieve a healthier and more equitable urban environment. As a result, they advocate for establishing healthy urban planning as a norm and the need for evidence-based leadership and political commitment to bring about change in urban planning practices.

Furthermore, they discuss the significance of identifying urban health problems and the need for appropriate actions to address them. Finally, they recognize the importance of innovative technologies and nature-based solutions in promoting public health, and suggest that programs and initiatives aimed at sustainable development will positively impact public health.

In conclusion, the participants provide valuable insights into the need for a holistic and proactive approach to urban planning that prioritizes public health. Furthermore, the emphasis on the role of nature-based solutions, innovative technologies, and sustainable development highlights the potential for positive change in the urban environment.

The findings provide a snapshot of the socioeconomic profile of the respondents. The gender distribution shows a slight skew towards males, while the age distribution highlights a significant presence of individuals aged between 36 and 55. The occupation distribution highlights a majority of respondents working in the public sector, with a notable presence of students and self-employed. Furthermore, the educational attainment of the sample reveals a relatively high level of education among respondents, with the majority holding a university degree.

The results from the first hypothesis-related variables provide strong evidence that implementing urban planning practices that promote improved sanitary systems has a significant positive impact on public health outcomes, as measured by residents’ satisfaction with the city’s response to the COVID-19 pandemic. Through various statistical tests, we have determined a statistically significant and strong association between residents’ perceptions of the city’s public health system’s accessibility and preparation for future epidemics and their satisfaction with the city’s response to the COVID-19 pandemic.

This highlights the importance of effective public health measures in fostering positive perceptions of government response during times of crisis. The city’s public health system’s preparation for future epidemics is critical in ensuring that residents feel satisfied with the city’s response to the COVID-19 pandemic. Our results suggest that the city’s public health system preparation after COVID-19 for future epidemics positively impacts the public health outcome.

The results from the second hypothesis-related variables, including chi-square tests and symmetric measures, indicate a statistically significant and strong association between satisfaction with the city’s efforts to promote a healthy lifestyle, the city’s management level for green spaces, and the importance of green space accessibility. These findings suggest that the city’s efforts to promote a healthy lifestyle, such as through accessible green spaces, significantly impact residents’ perceptions of their overall well-being. This highlights the crucial role that urban planning practices can play in fostering positive public health outcomes, particularly in the context of the ongoing COVID-19 pandemic and its impact on mental and physical health, which provides strong empirical evidence supporting the research, which posits that the implementation of urban planning practices that prioritize residents’ mental and physical well-being post-COVID-19 has a significant positive impact on public health outcomes.

The results from the third hypothesis-related variables provide evidence of a significant positive association between policy initiatives and programs that support sustainable development and improve the urban environment and their impact on public health outcomes. The use of chi-square tests and symmetric measures such as Kendall’s tau-b, Spearman correlation, and Pearson’s R all indicate a strong positive correlation between the studied variables. Specifically, the analysis shows a strong association between satisfaction with health and safety measures implemented in the city after COVID-19 and the city’s urban planning policies’ level of promoting health and well-being, as well as between satisfaction with health and safety measures implemented in the city after COVID-19 and the effectiveness of management in addressing public health concerns related to COVID-19. These findings suggest that cities prioritizing sustainable development and improving the urban environment through policy initiatives and programs positively impact public health outcomes, particularly during the COVID-19 pandemic.

The site visits investigation aimed to examine the public health situation in Algiers and the urban planning practices after the COVID-19 pandemic, as declared in the semi-structured interviews’ responses from two perspectives:

- Sanitary System and Epidemiology: Results revealed ongoing efforts in various domains of sanitary systems and epidemiology from many levels, such as the efforts to prevent sewage mishaps and maintain the sewage network, including operational measures to clean sewer systems, address malfunctioning systems, repairing existing breakdowns, enhancing the quality of potable water, upgrading the outdated pipelines, and maintaining its access points. The mitigation of toxic exposure risk is also ongoing in the city’s industrial zone. Waste collection and disposal are being handled by a private company and the government incorporating new technologies and sorting strategies, which supports that there is a positive effect of the pandemic on strengthening the link between urban planning and public health in Algiers.
- Healthy City, Management, and Green Spaces Implementation: The results suggest that efforts to improve and maintain green spaces are increasing. Nevertheless, disparities in the availability and accessibility of green spaces were identified across the city. The Bainam forest tree plantation initiative, which had suffered severe degradation in the past decade due to fires and natural conditions, is progressing well and is nearly complete. The launch of awareness campaigns in partnership with civil society, aimed at raising awareness about the role of citizens in preserving the environment and improving public health, was noted during the investigation. Furthermore, the transformation of the Oued S’mar public landfill into a public garden has been completed. These findings highlight the positive impact of the COVID-19 pandemic on the relationship between city management and public health outcomes.

The COVID-19 pandemic has shaped new behaviors in urban planning to promote public health. The study’s findings indicate ongoing efforts in various domains to improve public health conditions and address the challenges posed by the pandemic, even if it is still at the modest progress rate of 58% of the required degree, which sheds light on the gap between the macro health-oriented strategies and actual managerial practices and the need to change its strategies.

The document analysis of the PDAU (Master Plan of Algiers) highlighted the various challenges and opportunities faced by the city in its efforts to achieve sustainable urban planning practices. The city is grappling with issues such as urbanization, excessive consumption of natural resources, degradation of the environment, transportation problems, and economic structural weaknesses, such as a lack of diversification and infrastructure. Despite these challenges, the city has several advantages: its favorable geographical location, rich cultural and natural heritage, rich natural resources, and a young, dynamic population. The Master Plan of Algiers seeks to address these challenges through six pillars, including economic development, territorial cohesion, environment, territorial model, and governance, and through 82 key projects to improve the city’s infrastructure, housing, and public health. The SWOT matrix helped us to identify the strengths, weaknesses, opportunities, and threats of the urban planning document, which gave us a clear understanding of the actual current urban planning practices and allowed us to locate public health considerations in the city’s Physical Development and Urbanism plan (PDAU).

The city must prioritize sustainable models, including economic, environmental, and social models, and preserve and develop its agricultural sector. The urbanization of Algiers must be carefully planned so as not to compromise its agricultural dynamics; additionally, it should aim to respond to the needs and aspirations of its population.

According to the investigation done after the classification of the interview’s findings, we found that while the relevant laws, regulations, and plans related to sustainable development and environmental protection, such as the National Plan of Action for the Environment and Sustainable Development and the Town Orientation Law, were in place, their implementation and enforcement on-site were not always consistent. On the other hand, a new policy announced by the Algerian Ministry of Environment and Renewable Energy at the end of December 2022 has initiated, in conjunction with the healthcare sector, the creation of a report on the implementation of the Arab Strategy for Health and Environment 2017–2030.

In urban planning practices, it was observed that the importance of waste management was considered; however, a need for implementation and enforcement was identified. Moreover, we found that the formation and qualification of healthy city urban planners faced some difficulties as some things could have been improved in terms of sustainability and green city knowledge among the local urban administration.

The investigation on the implementation of the significant decisions, initiatives, and policies for enhancing public health after COVID-19 in Algeria revealed that current urban planning practices require improvement to align better with initiatives and policies aimed at promoting sustainable development and environmental protection.

On-site visits revealed positive progress in certain areas, such as transforming a public landfill into a public garden. Still, only 58% of the defined initiatives and decisions were applicable. The study also highlighted the recent policy announced by the Ministry of Environment and Renewable Energy, which aims to create a report on the implementation of the Arab Strategy for Health and Environment and a new regulation for the management, protection, and development of green spaces.

In order to summarize and clarify the findings from all the discussion parts, we gathered in the table below all the study phases that contributed to either supporting or rejecting the research hypotheses (Table 5).

## 6. Conclusions

The relationship between urban planning practices and public health is crucial in shaping individuals’ well-being in urban spaces. The findings of this study, obtained through semi-structured interviews and a survey, emphasized the role of the COVID-19 pandemic in the shaping of a comprehensive, health-centric approach to city design, improved governance and management practices, community involvement, and political commitment to prioritize health in urban planning. The results showed a strong correlation between implementing urban planning practices prioritizing public health and residents’ satisfaction with the city’s response to the COVID-19 pandemic. The need for a proactive, preventive approach to addressing public health and environmental issues was highlighted rather than it being a reactive one. It is imperative that policymakers invest in advanced and effective sewage treatment technologies, upgrade existing sewage infrastructure, incorporate health considerations into urban planning policies, and promote sustainable development and eco-friendly practices. In addition, the availability and accessibility of green spaces and the implementation of healthy-urban planning concepts in universities should also be prioritized.

The findings emphasize the critical importance of sanitary systems, healthy city management and governance, and health integration in urban planning policy. In addition, the need for stakeholders to work together towards a healthier and more equitable urban environment was also emphasized. These insights highlight the significance of considering public health as a priority in urban planning practices and the need for a comprehensive long-term approach that considers the needs of both individuals and communities. In conclusion, urban planning practices are vital in promoting public health and ensuring the well-being of individuals living in urban spaces. Therefore, all stakeholders, including policymakers, city planners, and the general public, must work towards achieving a healthier and more equitable urban environment.

## Figures and Tables

**Table 1:** Theme 1: sanitary elements affecting public health in urban space—results interpretation.

Sub-Theme	Main Declarations	Interpretation
Proper sewage system	I#9: “It is the responsibility of the conservation and public health sectors to prevent disasters linked to sewage overflowing or pollution”. I#1: “SEAAL (Algiers water and sanitation company) is doing quite good work in collecting and purification”. I#6: “Things become complicated from early December to late march. It is the rain and floods season, and because of the old, degraded condition of pipes and maintenance holes, specifically in the ancient part of the city… it always requires interventions to solve it; rains bring persistent urban challenges”.	Interviewees emphasize the need for joint efforts between conservation and public health sectors to prevent sewage-linked disasters and pollution. They praise the work of SEAAL in the collection and purification process and shed light on the challenges during the rain and floods season, particularly in the ancient part, due to the old and degraded condition of pipes and maintenance holes.
Risk of toxic contamination in cities	I#10: “I believe that the risk of exposure to toxic materials and chemical mixtures in the urban environment on the local level is relatively low”. I#2: “Unfortunately, the fast-going act of the industrial zones of the northern wilayas of Algeria turns the mission of checking toxic waste data for control agencies even more difficult”.	Interviewees discuss the issue of exposure to toxic materials and chemicals in the urban environment. They noted that the rapid development of industrial zones in the northern region of Algeria makes it difficult for control agencies to monitor toxic waste data.
Waste management practices in urban space	I#7: “On the local level, the waste management field used to suffer from the lack of availability of human and material resources, which lasted until four years ago when the municipal council took on the responsibility of signing a contract with a private company (Extranet) along with the government capabilities for a better household waste collecting and disposal operating: double rounds, better efficiency, and recycling strategy”. I#12: “we understand the emphasis of waste management for a healthy city, and we aim to stay updated to the emerging waste management technologies and their associated effects on the public health”.	The interviewees highlight the importance of waste management in ensuring public health in urban areas. They emphasize the role of the local government and private sector in improving waste collection and disposal through the use of efficient systems and recycling strategies, and stress the importance of staying informed about new waste management technologies and their impact on public health. Both quotes showcase a commitment to preventative strategies in urban planning to address public health concerns.
Experience gained from the COVID-19 pandemic in urban planning	I#6: “we learned from the COVID-19 pandemic that education alone cannot fix the problem, neither can medicine… we now have chronic diseases associated with the habitable environment, just like respiratory diseases, obesity, and others, so we need to keep working on the same path to achieve a city that can fight pandemics and infectious illnesses and be an aid to the medical profession to make their mission easier and less complicated”. I#11: “COVID-19 pandemic demonstrated to us how venerable we can be and how non-prepared our cities are to face such a circumstance, but we took a precious experience from the catastrophe in what concerns crisis management and pandemics fighting from punctual levels such as hospitals and healthcare facilities”.	The interviewees underline the importance of implementing innovative and holistic preventive strategies in urban planning to address public health concerns and address the vulnerability exposed by the COVID-19 pandemic. Additionally, they discuss the valuable experience gained from the pandemic in crisis management and fighting pandemics at the local level, including hospitals and healthcare facilities.
New preventive strategy in urban planning	I#1: “The COVID-19 pandemic has highlighted the need for innovative preventive strategies in urban planning to ensure public health and safety in our cities”. I#5: “The new normal has made it imperative for urban planners to reconsider traditional planning approaches and embrace a holistic, health-centric approach to city design”.	The interviewees emphasize the importance of innovative solutions to ensure public health and safety in urban areas after the pandemic. However, the interviewees also acknowledge the need for a shift in traditional planning approaches towards a more holistic, health-centric approach to city design to reflect the new normal.

**Table 2:** Theme 2: management, governance, and leadership skills for the identification of the healthy city concept—results and interpretation.

Sub-Theme	Main Declarations	Interpretation
Managerial practices	I#1: “A key factor in the success of the healthy city movement is good city governance and right managerial decisions”. I#5: “Healthy urban planning is always related to good city governance and optimum managerial choices, along with taking into consideration the realistic factors affecting the optimistic aspirations”.	The interviewees emphasized the importance of effective city governance and sound decision-making in creating a healthy urban environment. They highlight the relationship between healthy urban planning and good governance, emphasizing that realistic factors must be considered to achieve positive outcomes.
Gap between macro health-oriented strategies and basic managerial techniques in the city	I#7: “The challenge in bridging the gap between macro health-oriented strategies and basic managerial techniques in the city lies in the lack of integration between the two approaches”. I#9: “The discrepancy between macro health-oriented strategies and basic managerial techniques in the wilaya highlights the need for a more comprehensive and coordinated approach to urban planning and public health”.	The interviewees highlight the challenge of aligning macro health strategies with local urban planning practices and emphasize the need for a more integrated approach to improve urban public health outcomes. They underline the importance of harmonious coordination between health-oriented strategies and managerial techniques in urban planning.
Fragmentation issues between different sectors	I#2: “It is clear that there is a problem of fragmentation when it comes to collaboration between the two sectors to achieve the desired aims of healthy cities”. I#12: “To achieve the healthy city goals, many efforts should be granted, and official collaborations of vertical levels of governance are needed. The efforts must include clear plans, budgets, and continuity mindset while applying them”.	The interviewees suggest a disconnection between macro-level health strategies and the city’s practical, day-to-day, management techniques. This disconnection leads to a fragmentation in collaboration between the two sectors, which hinders the achievement of the goal of a healthy city. To overcome this challenge, it is necessary to implement an official collaboration between different levels of governance, with clear plans, budgets, and a continuous mindset in implementing these plans.
formation and qualification of healthy urban planners	I#9: “I am afraid we still lack the right expertise and qualification in sustainability and green cities at the level of local urban administrations, which represents a fence limiting the good practices here”. I#3: “The concept of healthy urban planning is already emerging in our universities… fresh graduates are well formed in what concerns the public health implementation in urban planning phases as they proved this in several projects in the last three years either as newly recruited engineers or even as trainees”. I#5: “The good qualification of managers and urbanists in terms of healthy planning is a key factor in achieving the healthy city objectives from the managerial level”.	The interviewees had mixed views about the challenge of creating a harmonious relationship between macro health-oriented strategies and local managerial techniques in urban planning due to the need for more integration between the two approaches. They expressed the need for increased expertise and qualification in sustainability and green city practices among local urban administrators. On the other hand, healthy urban planning is gaining traction in universities, with fresh graduates well-equipped to implement public health in urban planning projects. Therefore, the qualification of urban planners in healthy planning is crucial in achieving healthy city objectives.
Implication of public community in the healthy city goals	I#8: “The healthy city project is applied to many cities in the north, middle, and even in the Sahara area of Algeria with some varieties in geographical and environmental elements as well as the nature of the urban texture in each city…. We mention one of some successful examples, Tizi Ouzou, which received the healthiest city reward twice this year and last year. However, it would not be possible without the union of efforts between the government and citizens of the city”. I#4: “Regarding implementing the healthy city concept and common efforts between community and government, the sustainable healthy concept has not yet been applied correctly. First, there needs to be a degree of awareness. Then the citizens should already feel a certain level of healthiness in their city to initiate the enhancement process”.	The interviewees highlight the challenge of integrating macro health-oriented strategies with basic managerial techniques in cities and the need to integrate the two approaches. Tizi Ouzou is mentioned as a successful example of a healthy city, highlighting the importance of the union of efforts between the government and citizens. Implementing the healthy city concept requires awareness and a sense of healthiness within the city’s community to initiate the enhancement process.

Source: Authors.

**Table 3:** Theme 3: integrating health concepts in the urban planning process—results and interpretation sub-theme.

	Main Declarations	Interpretation
The current state of urban planning from a healthy perspective	I#10: “We need to admit that we are still far from the ideal healthy city requirements for the moment… it is obvious that the attempt to improve the situation on both levels is ongoing”.	The interviewee highlighted that the challenge of implementing healthy urban planning lies in the lack of integration between macro health-oriented strategies and basic managerial techniques in the city.
Urban health problems’ identification	I#7: “We need to identify the illness first to take actions that can improve health in the urban space. The identification process could be rigorous and expensive in both time and budget, but an absolute urgency to solve the existing issues and avoid future complications”.	The interviewee mentioned that the challenge in implementing healthy urban planning is due to the disconnect between high-level health strategies and practical management techniques in the city.
Need for change in planning and policy	I#3: “To develop a shared prevision for a healthier and more equitable urban place, we need a serious commitment from the different stakeholders, partners, and policy decisions” I#3: “Organizational attempts are needed to establish healthy urban as a norm for planning practices”. I#12: “Building environment and public health professions require some more proof of leadership and political commitment” (I#12) I#5: “We need to start planning our urban habitable space more healthily, especially roads, maybe implementing more walkable roads near habitats with special design that encourages citizens to walk more frequently”.	Changes are needed in urban planning and policy-making to develop a healthier and more equitable urban place. Therefore, to achieve a healthier and fairer urban environment, there must be a strong commitment from all stakeholders and policymakers, including efforts to establish healthy urban planning as a norm, leadership and political support from environment and health professionals, and a focus on designing walkable roads to encourage healthy habits.

Source: Authors.

**Table 4:** On-site investigation steps.

Theme	Sub-Theme	Initiative/Decision	Location in Algiers	Type of Investigation	Status and Percentage
Sanitary system and epidemiology	Sewage and wastewater	Prevention of disasters linked to sewage overflowing or pollution by maintaining the sewage system.	Chéraga Kouba Ben Aknoun Casbah Beb El Oued	✓ Checking the VRD plans ✓ Observation	In progress: 40%
		ONA (National Sanitation Office): wastewater treatment plant under construction as part of a multi-year national action plan.	Dar El Beida	✓ Construction site observation ✓ Project manager declaration	In progress: 20%
	Quality of water	SEAAL: improvement of the quality of water produced and distributed at the level of the Wilayas of Algiers.	Kouba	✓ checking the results of periodic analyzes of the drinking water (pH, total dissolved solids, heavy metals, presence of bacteria and virus’s tests)	Compliant with standards: 100%
	Rain and floods season	Interventions to solve old, degraded pipes and maintenance holes in ancient part of the city.	Casbah Chéraga Draria Hussein Dey	✓ Observation of pipes in a rainy day	In progress: 60%
	Risk of exposure to toxic materials	Minimizing the risk of exposure to toxic materials and chemical mixtures in urban environment on local level.	Reghaia lake Oued El Harrach Rouiba	✓ Visits to 16 company, 5 of them are suspended until acquiring purification stations for toxic and dangerous liquids.	In progress: 30%
Healthy city, management, and green spaces implementation.	Better use of land	Transforming Oued S’mar public landfill into a public garden	Oued S’mar	✓ Observation of a 45 Ha public garden for relaxation	Done 100%
	Green spaces management	Renovation of green spaces in all the districts of Algiers	Chéraga Draria Hussein Dey Beb El Oued Kouba Mouhamadia	✓ Observation	In progress: 20%
		Bainam forest tree plantation initiative	Bainam	✓ Observation	In progress: 90%
	Waste management	Waste collecting and disposal operating with contract with private company and government capabilities	Beb El Oued Casbah	✓ Citizen’s confirmation ✓ Observation	In progress: 60%
		Using new technologies in collection	Chéraga Draria Hussein Dey Beb El Oued Kouba	✓ Citizen’s confirmation ✓ Observation	In progress: 30%

Source: authors.

**Table 5:** Hypotheses supporting status according to study phases. Source: authors.

Hypothesis	Interviews	Survey	PDAU Analysis	Site Visits
Hypothesis 1	Supported	Supported	No contribution	Supported
Hypothesis 2	Supported	Supported	Supported	Supported
Hypothesis 3	Supported	Supported	Supported	No contribution

## Data Availability

Some or all data, models, or codes that support the findings of this study are available from the corresponding authors upon reasonable request.

## Appendix A. The Interview Guide

Introduction:

Welcome. This interview is a part of our research study titled “From Sanitary Systems to Policy Implications: Understanding the Impact of Urban Planning Practices on Public Health Post COVID-19”. The study aims to understand the impact of urban planning practices on public health, focusing on northeastern Algeria and explicitly addressing the impact of the sanitary system, urban green spaces, and policy on public health outcomes. Our goal is to provide clear guidance for engineers and policymakers to adjust current management practices to improve public health outcomes, especially in the COVID-19 framework.

We will be asking you a series of questions about your professional experience and familiarity with healthy urban planning concepts, as well as specific themes related to sanitary systems, urban green spaces, and the role of policy for healthy cities. Participation in this study is greatly appreciated, and all information provided will be kept anonymous and confidential. However, this interview will be recorded for research purposes.

Questions:

1. Can you tell me your age and occupation?
2. How many years of experience in urban planning/ Public health practices?
3. How familiar are you with the concept of healthy urban planning?
4. Can you describe your professional experience and background concerning urban and public planning practices?

Sanitary System:

5. What are some of the current challenges or problems you have encountered regarding your city’s sewage system, toxicity, trash, or epidemiology?
6. Can you explain the current condition of the sewage system, collection, paths, and treatment in your city?
7. How are sanitary system problems identified in your city?
8. What are the problems faced and suggested solutions for better public health circumstances in the city?
9. How do you see the management and governance of the city impacting public health and well-being?
10. Can you say something about the gap between macro health-oriented strategies and basic managerial techniques in the city?
11. How do fragmentation issues between different sectors affect the promotion of a healthy city?

Urban Green Spaces:

12. How does the formation and qualification of healthy urban planners impact the implementation of new healthy city concepts in urbanism curriculums?
13. How important do you believe public community involvement is in achieving healthy city goals?
14. Can you say something about the availability and accessibility of urban green spaces in your city?
15. How do urban green spaces impact citizens’ mental and physical health?

Policy:

16. How do you identify urban health problems in your city?
17. How do you integrate health concepts into the urban planning process?
18. How should urban planning policies be changed to promote healthier cities, especially after COVID-19?
19. Can you explain the current state of urban planning policy from a healthy perspective?
20. How do you involve other stakeholders in this process?
21. How do you ensure sustainable development is achieved through this process?
22. How do you measure the success of this process within your position?
23. What are the most important initiatives and policies in your wilaya concerning healthy urban planning?
24. What are some of the barriers you have encountered in implementing healthy urban planning?
25. What are some of your suggestions for future development in healthy urban planning?

That was all. Thank you for your time and assistance in this important study.

## Appendix B. The Questionnaire

Thank you for taking the time to participate in this survey. This questionnaire aims to assess the applicability of initiatives and decisions related to public health, urban planning, and management in the city of Algiers after the COVID-19 pandemic.

Your participation is entirely voluntary and confidential. Your answers will be used for research purposes only and kept strictly confidential. The survey consists of 18 questions divided into three parts: demographic information, sanitary system and epidemiology; healthy city, management, and green spaces implementation; and health integration in urban planning policy. Please note that your participation in this survey is entirely voluntary and confidential. The questionnaire is designed as a Likert scale, where 5 represents “strongly agree”, 4 represents “agree”, 3 represents “neutral”, 2 represents “disagree”, and 1 represents “strongly disagree”.

Please answer all questions as honestly and accurately as possible. Your input is greatly appreciated.

Demographic Information:

1. Gender:
  - Male
  - Female
2. Age:
  - Specify
3. Occupation:
  - Student
  - Public Sector Employee
  - Private Sector Employee
  - Self-Employed
  - Retired
4. Education Level:
  - Less than High School
  - High School
  - University
  - Post-Graduate

Part 1: Sanitary System and Epidemiology

5. How satisfied are you with the city’s response to the COVID-19 pandemic?
  - Strongly dissatisfied
  - Dissatisfied
  - Neutral
  - Satisfied
  - Strongly satisfied
6. How well do you think the city’s public health system is prepared for future epidemics?
  - Strongly unprepared
  - Unprepared
  - Neutral
  - Prepared
  - Strongly prepared
7. Do you think the city’s public health system is accessible to all residents?
  - Strongly disagree
  - Disagree
  - Neutral
  - Agree
  - Strongly agree

Part 2: Healthy City, Management, and Green Spaces Implementation

8. How satisfied are you with the city’s efforts to promote a healthy lifestyle?
  - Strongly dissatisfied
  - Dissatisfied
  - Neutral
  - Satisfied
  - Strongly satisfied
9. How well do you think the city’s management of green spaces is?
  - Poorly
  - Somewhat poorly
  - Neutral
  - Somewhat well
  - Well
10. How important are the city’s accessible green spaces?
  - Not at all important
  - Somewhat unimportant
  - Neutral
  - Somewhat important
  - Very important

Part 3: Health Integration in Urban Planning Policy

11. How well do the city’s urban planning policies promote health and well-being?
  - Poorly
  - Somewhat poorly
  - Neutral
  - Somewhat well
  - Well
12. How satisfied are you with the city’s efforts to integrate health into urban planning decisions?
  - Strongly dissatisfied
  - Dissatisfied
  - Neutral
  - Satisfied
  - Strongly satisfied
13. How satisfied are you with the overall health and safety measures implemented in Algiers after COVID-19?
  - Strongly Disagree
  - Disagree
  - Neutral
  - Agree
  - Strongly Agree
14. How effective do you believe the city’s management has been in addressing public health concerns related to COVID-19?
  - Strongly Disagree
  - Disagree
  - Neutral
  - Agree
  - Strongly Agree
